# Fine structure of the silk spinning system in the caddisworm, *Hydatophylax nigrovittatus* (Trichoptera: Limnephilidae)

**DOI:** 10.1186/s42649-020-00036-5

**Published:** 2020-08-06

**Authors:** Hyo-Jeong Kim, Yan Sun, Myung-Jin Moon

**Affiliations:** Department of Biological Sciences, Dankook University, 119 Dandae-ro, Cheonan, 31116 South Korea

**Keywords:** Fine structure, Silk, Caddisworm, *Hydatophylax nigrovittatus*

## Abstract

Silk is produced by a variety of insects, but only silk made by terrestrial arthropods has been examined in detail. To fill the gap, this study was designed to understand the silk spinning system of aquatic insect. The larvae of caddis flies, *Hydatophylax nigrovittatus* produce silk through a pair of labial silk glands and use raw silk to protect themselves in the aquatic environment. The result of this study clearly shows that although silk fibers are made under aquatic conditions, the cellular silk production system is quite similar to that of terrestrial arthropods. Typically, silk production in caddisworm has been achieved by two independent processes in the silk glands. This includes the synthesis of silk fibroin in the posterior region, the production of adhesive glycoproteins in the anterior region, which are ultimately accumulated into functional silk dope and converted to a silk ribbon coated with gluey substances. At the cellular level, each substance of fibroin and glycoprotein is specifically synthesized at different locations, and then transported from the rough ER to the Golgi apparatus as transport vesicles, respectively. Thereafter, the secretory vesicles gradually increase in size by vesicular fusion, forming larger secretory granules containing specific proteins. It was found that these granules eventually migrate to the apical membrane and are exocytosed into the lumen by a mechanism of merocrine secretion.

## Introduction

Silk production occurs in bees, wasps, ants (Hymenoptera), silverfish (Zygentoma), mayflies (Ephemeroptera), thrips (Thysanoptera), leafhoppers (Hemiptera), beetles (Coleoptera), lacewings (Neuroptera), fleas (Siphonaptera), flies and midges (Diptera) (Yang and Merritt 2003; Sehnal and Sutherland 2008; Sutherland et al. 2010). Although silk is mainly produced by the larvae of insects undergoing complete metamorphosis, some insects, such as raspy crickets (Orthoptera) (Walker et al. 2012) and web spinners (Embioptera) (Büsse et al. 2019) also produce silk throughout their lives.

Silks are also produced underwater by several other aquatic insects, but only the silks produced by terrestrial arthropods including a few insects (Sasaki et al. 1981; Akai 1984; Sehnal and Akai 1990; Yang and Merritt 2003; Walker et al. 2012; Büsse et al. 2019) and some spiders (Tillinghast and Townley 1986; O'Brien et al., 1998; Moon and Tillinghast 2002; Park and Moon 2014) have been examined in detail, and only the silk of moth caterpillars has been used for textile manufacturing (Sehnal and Akai 1990; Sehnal and Sutherland 2008). Recently it has been reported a small shrimp-like crustacean that produces the adhesive underwater silk for use as a binding material in building its undersea home (Kronenberger et al. 2012).

The caddisflies are a group of insects with aquatic larvae and terrestrial adults. The aquatic larvae are widely distributed in freshwater habitats throughout the world in an order of Trichoptera (Wiggins 2004). They are related to Lepidoptera the order that includes moths and butterflies that spin dry silk. Aquatic caddisflies and terrestrial butterflies and moths diverged from a common silk-spinning ancestor about 250 million years ago (Wiggins 2004). The aquatic caddisfly larva of many species produce adhesive silk from the silk glands on the lower lip (labium), and use silk to construct protective shelters or a funnel shaped webs in running water to catch food (Sehnal and Sutherland 2008).

The form of trichopteran silk has been described as flat ribbons, a homogeneous glue-like sheet, and a very fine, irregular reticulation embedded in an amorphous layer (Tindall 1960). Recently, the trichopteran silk has been focused as a medical bioadhesive for sticking to wet tissues, since their adhesive is able to bond to a wide range of surfaces underwater both of organic and inorganic (Tszydel et al. 2015). The caddisfly silk is a fiber made of large fibroin proteins, and that is a key factor in making silk underwater because of the electrical charges (Stewart and Wang 2010).

Physically, two different processes of silk-producing systems are reported among the different species of arthropod animals: terrestrial and aquatic silk productions (Rudall and Kenchington 1971; Craig 1997; Sutherland et al. 2010; Ashton et al. 2016). Although both types of silks can be produced along its sophisticated process through a sequential pathway from silk gland, most of our recent knowledge of silk producing system are dependent on those revealed from the terrestrial animals including silkworms (Akai and Kobayash 1965; Sasaki et al. 1981; Akai 1984; Sehnal and Akai 1990) or spiders (Kovoor 1987; Weiskopf et al. 1996; Vollrath and Knight 2001; Moon and Tillinghast 2004; Moon and Park 2008; Crew and Opell 2006; Moon 2018).

However, there are few studies on aquatic silk, and little is known about the ultrastructural characteristics of trichopteran silk gland itself. Except for the study on the silk from the sand-grain case of *Olinga feredayi* (Rudall and Kenchington 1971) and several other brief studies, the histological and fine structural study of a trichopteran silk gland have been nearly neglected. Therefore, this experiment was initiated to reveal the fine structural aspects of the silk producing system of larval stage of the caddisfly *Hydatophylax nigrovittatus* with light and electron microscopes.

## Materials & methods

The larvae of caddisworm *Hydatophylax nigrovittatus* were collected in early May in the upper reaches of Guangdek mountain valley, Cheonan, Chungcheongnam-do, Korea. For histologic preparation, specimens were dissected under a dissecting light microscope in a drop of insect Ringer’s solution consisting of 128 mM NaCl, 18 mM CaCl_2_, 1.3 mM KCl, 2.3 mM NaHCO_3_, pH 7.4 (Moon and Yang 2014).

Both of spinnerets and silk glands were gently removed and fixed in alcoholic Bouin’s solution, and dehydrated through an ethanol series from 30 to l00% (30 min at each concentration, with one repeat at 100% ethanol). After dehydration, the specimens were transferred to xylene for clearing at room temperature to 60 °C, and they were embedded with Paraplast embedding medium (Fisher Scientific Co., Pittsburgh, Pa, USA) at 60 °C. The sections were cut with a thickness of approximately 5 μm using a rotary microtome, Leica (RM-2135, Leica, Nussloch, Germany) and they were stained with hematoxylin and eosin (H & E) solutions.

For scanning electron microscopic (SEM) experiment, the specimens were prefixed in a mixture of 2% paraformaldehyde and 2.5% glutaraldehyde buffered with 0.1 M phosphate buffer at pH 7.4. Postfixation was performed with 1% osmium tetroxide in the same buffer and washed several times in 0.1 M phosphate buffer. Following fixation, the specimens were carefully dehydrated in ascending concentrations of ethanol from 30 to l00%, and then either critical point-dried or transferred to hexamethyldisilazane (HMDS) for air-dry (Kim et al. 2016). All samples were coated to a thickness of approximately 20 nm with gold-palladium alloy using a sputter coater and examined on a Hitachi (S-4300, Hitachi Ltd., Tokyo, Japan) field emission scanning electron microscopy (FESEM) operated with an accelerating voltages of 5–15 kV.

For transmission electron microscopic (TEM) experiment, both of spinnerets and silk glands were fixed and dehydrated according to the same protocol for SEM experiment. The specimens were then embedded in Poly/Bed 812-Araldite medium (Polysciences Inc., Warrington, PA, USA) via propylene oxide. Semi-thin sections, 0.5–1.0 μm thick, were obtained using a Richert Ultracut R (Leica, Nussloch, Germany) and were stained with 1% toluidine blue (dissolved in 1% borax). Microscopic images were photographed using Zeiss Axiophot microscope (Carl Zeiss, Jena, Germany) coupled with a Motic digital imaging system (Motic Instruments Inc., Richmond, Canada).

Ultrathin sections were obtained using an Ultra 45° diamond knife (Diatome, Hartfield, PA, USA), and were double stained with uranyl acetate, followed by lead citrate. After these treatments, the sections were examined with a JEM 2100 Plus transmission electron microscope (JEOL, Tokyo, Japan) of the Korea Basic Science Institute (KBSI) at Ochang, Chungbuk, South Korea at accelerating voltage of 100 kV.

## Results

**Fig. 1:**
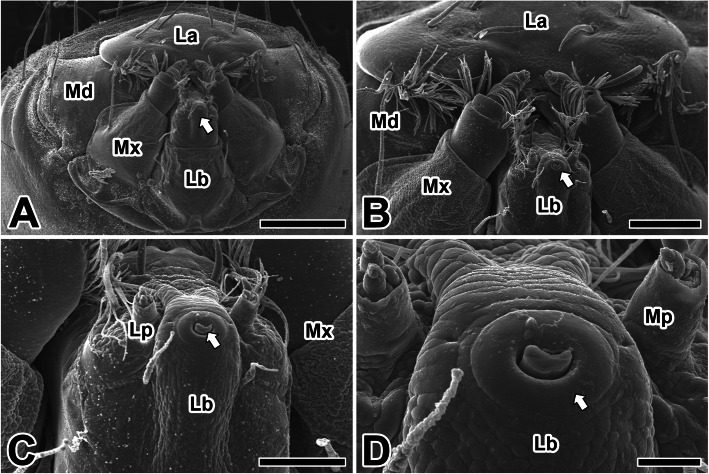
**a**-**d** Scanning electron micrographs of the mouthparts in the caddisworm *H. nigrovittatus.* Arrow indicates spinneret of caddisworm, Md: mandible, Mx: maxilla, La: labrum, Lb: labium, Lp: labial palp, Mp: maxillary palp. Scale bars indicate 500 μm (**a**), 200 μm (**b**), 100 μm (**c**), and 25 μm (**d**), respectively

The larva of caddisfly *H. nigrovittatus* has an armored head and a chewing type of mouthpart, which consists of a labrum, a labium, and a pair of mandibles and maxillae. Among them, the mandible is the largest and most powerful mouthpart of the caddisworm. Below the mandible is the maxillae, and at the upper side of the maxilla there is the maxillary palp (Fig. 1a).

The labium is located in the lower region of the mouthpart and fuses to the midline, forming the lower lip. A pair of labial palpi are visible on either side of the labium (Fig. 1b). The adhesive silk ribbon of the caddisfly larvae is spun out of the cuticular spinneret located at the apical end of the labium (Fig. 1c). Since the silk material produced by a pair of silk glands converges to common duct of the spinneret, the silk fibers has a morphology of a double ribbon with a seam the long way (Fig. 1d).

**Fig. 2:**
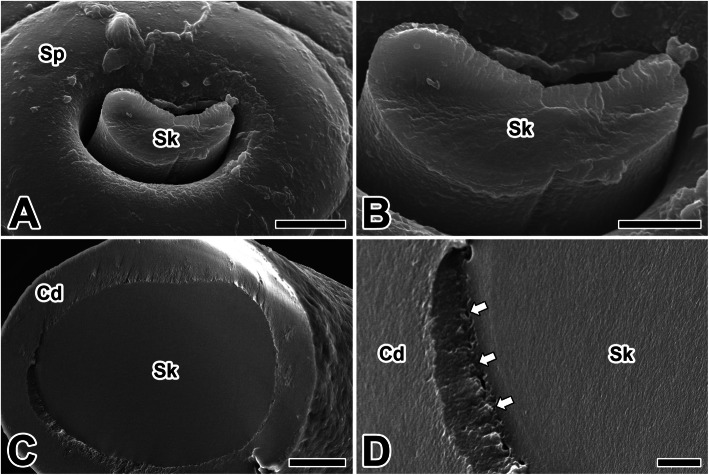
**a**-**d** Scanning electron micrographs of the spinneret (Sp) and silk fiber (Sk) of the caddisworm *H. nigrovittatus.* Sp: spinneret. Arrows indicate the marginal area between the cuticular duct and luminal silk material. Scale bars indicate 25 μm (**c**), 10 μm (**a**), and 5 μm (**b, d**), respectively

The anterior portion of each silk gland passed through the head, and they are fused at a spinneret below the mouthparts. Since the aquatic caddisfly larvae spin adhesive silk fibers for underwater construction of protective composite silken cases, the silk fibers produced from the spinneret have the appearance of flat fibrous ribbon embedded in the adhesive coat layer (Fig. 2a). Scanning electron microscopic examination clearly shows a distinct peripheral coating layer on a core fiber (Fig. 2b). Caddisworm silk fibers are drawn from silk precursors stored in the posterior region of the silk gland. However, the process of curing the silk fiber begins in the cuticular duct leading to the spinneret (Fig. 2c, d).

**Fig. 3:**
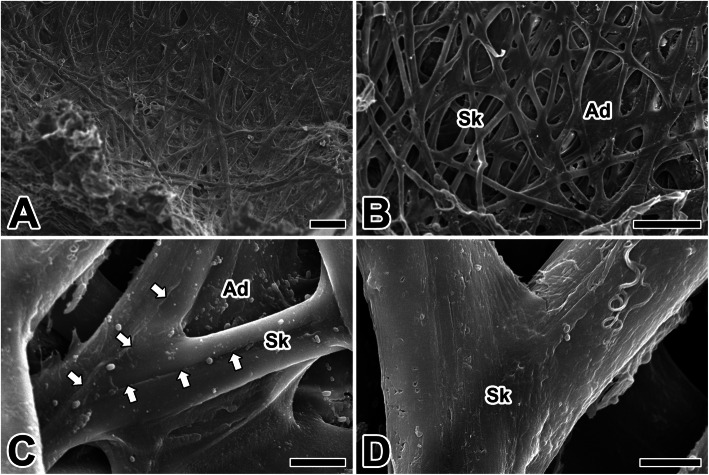
**a**-**d** Scanning electron micrographs of the composite silken case produced by the silk ribbons (Sk) and adhesive coats (Ad) of the caddisworm *H. nigrovittatus.* Arrows indicate the midline traces of fibrillar fusion. Scale bars indicate 100 μm (**a, b**), 10 μm (**c**), and 5 μm (**d**), respectively

The caddisworm builds its silken case by producing with a long and continuous fiber. The silk ribbons are produced to form a loose network of silken cases. Below this mesh, a dense buff pad is formed. Both the inner and outer surfaces are made of a loose network of silk ribbons, but the outer surface is denser and more opaque and has an adhesive coat layer (Fig. 3a). This difference may be due to the presence of a covering layers containing a gluey substance in the complex structure of the silk ribbon, since some fibers are typically visible even in the coated constructions (Fig. 3b).

The secretions from a pair of silk glands come from a single tube of spinneret in the mouthpart. When exposed to a water environment, it cures to form a twin filament of silk material (Fig. 3c). Apparently, the presence of some divulged fibers clearly indicates that the individual silk fibers are subsequently mixed after being extruded from the spinneret (Fig. 3d).

**Fig. 4:**
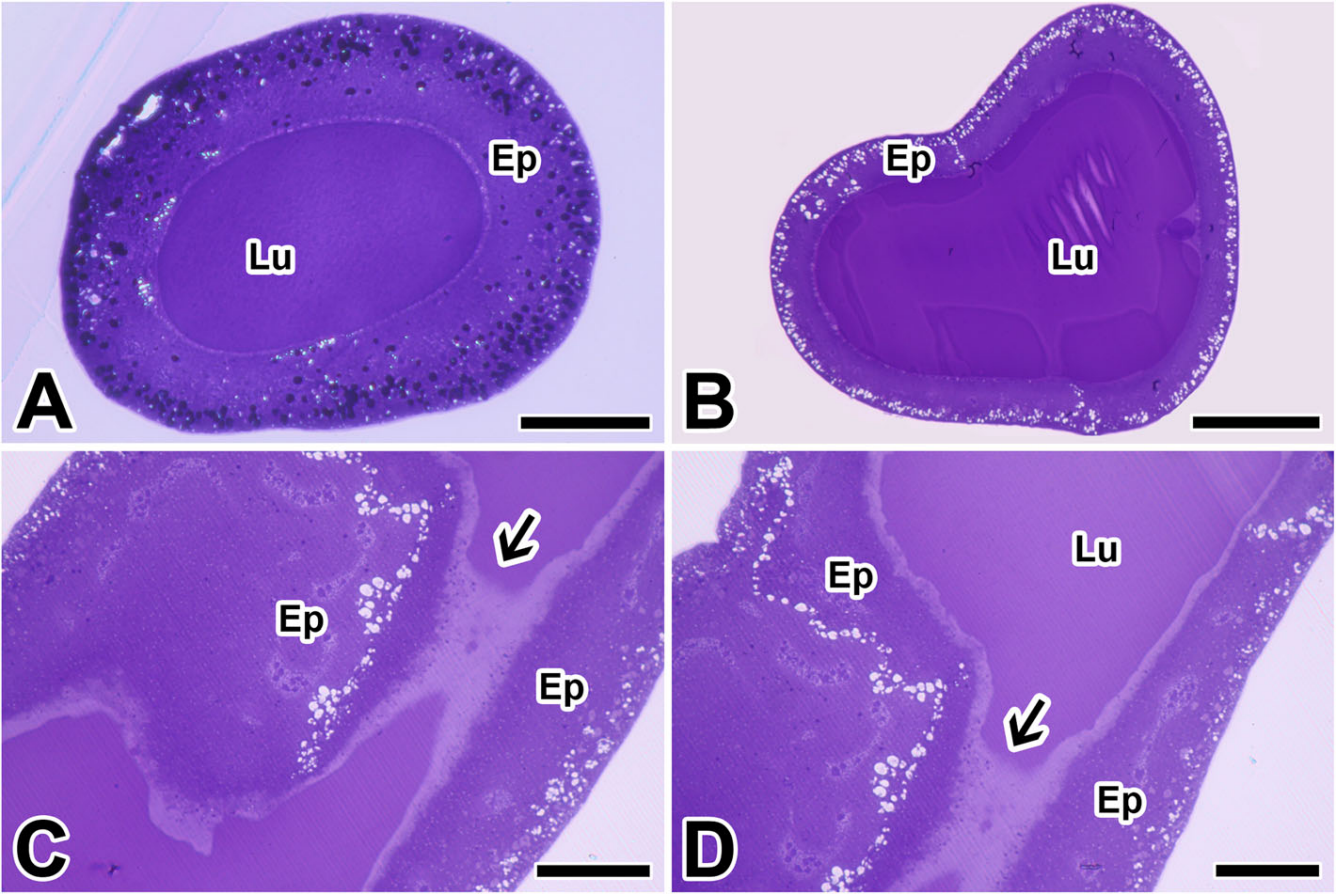
Cross section of the silk gland in the caddisworm *H. nigrovittatus.***a**, **b** Cross section of anterior (**a**) and posterior (**b**) silk gland showing glandular epithelium (Ep) and internal lumen (Lu). **c**, **d** Longitudinal section of the silk gland illustrating an annular narrowing (arrows) at the transition continuous with the cuticular lining of the anterior silk gland. All scale bar indicates 100 μm

By light microscopic observation, silk gland embedded in epoxy resin shows two basic regions which composed of outer glandular epithelium and inner lumen containing silk dope. The silk precursors in the lumen stained with toluidine blue occur as an uniform density with dark globular inclusions (Fig. 4a, b). The transition from posterior to anterior gland shows the annular narrowing continuous with the cuticular lining. Therefore, there is a decrease in the radius of the silk gland lumen, and inclusions in the luminal contents become elongated upon passage into the anterior lumen (Fig. 4c). Since silk fibers begin to form at a cuticular narrowing leading to the spinneret, the silk comprises a thin adhesive peripheral coating on a core fiber (Fig. 4d).

**Fig. 5:**
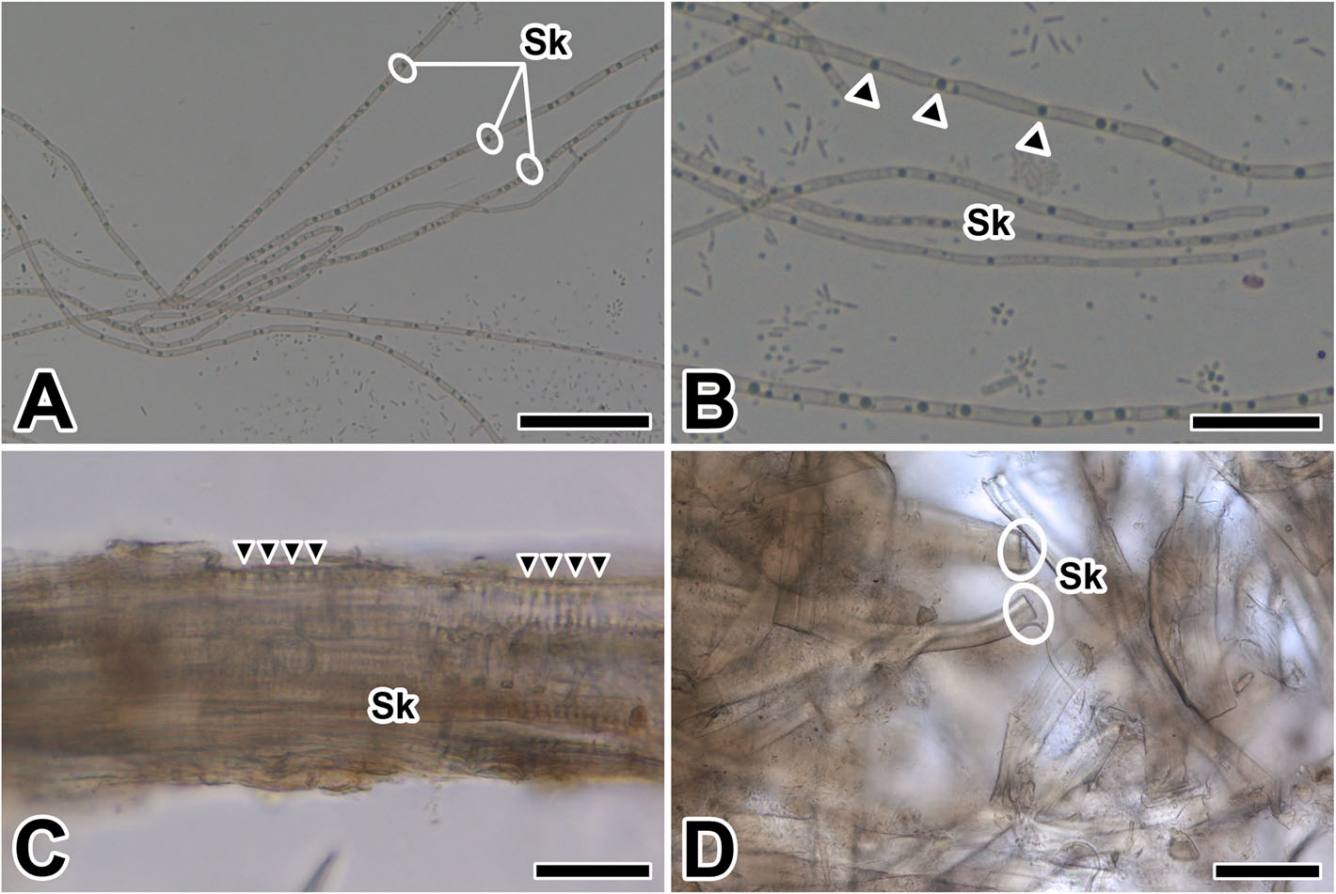
Photo micrographs of the silk fibers in the caddisworm *H. nigrovittatus.***a**, **b** Each silk fiber (Sk) shows peculiar banding pattern as alternating dark (arrowheads) and light bands. **c**, **d** The alignment of fibers in the silken case causes the entire silk ribbon to appear striated (arrowheads). Scale bars indicate 50 μm (**a**), 25 μm (**b**), 10 μm (**c**), and 5 μm (**d**), respectively

Finally, silk fibers of the caddisworm begin to spin from the spinneret with a thin adhesive peripheral coating on a core fiber. The products of two silk glands converge there, so the extruded adhesive looks like a ribbon (Fig. 5a). Each silk fiber has approximately 1 μm in diameter and shows peculiar banding pattern as alternating dark and light bands with average 15 μm intervals (Fig. 5b). To increase the tension of silk ribbon, each fiber has a compactly striated (pleated) pattern. The alignment of fibers in the silken case causes the entire silk ribbon to appear striated or banded (Fig. 5c). As seen in a loose network of silken case, each silk ribbon is surrounded with a gluey covering material. The fibrillar core of caddisworm silk is coated with an adhesive layer that fuses paired fibers into a single ribbon and bonds the silken case (Fig. 5d).

**Fig. 6:**
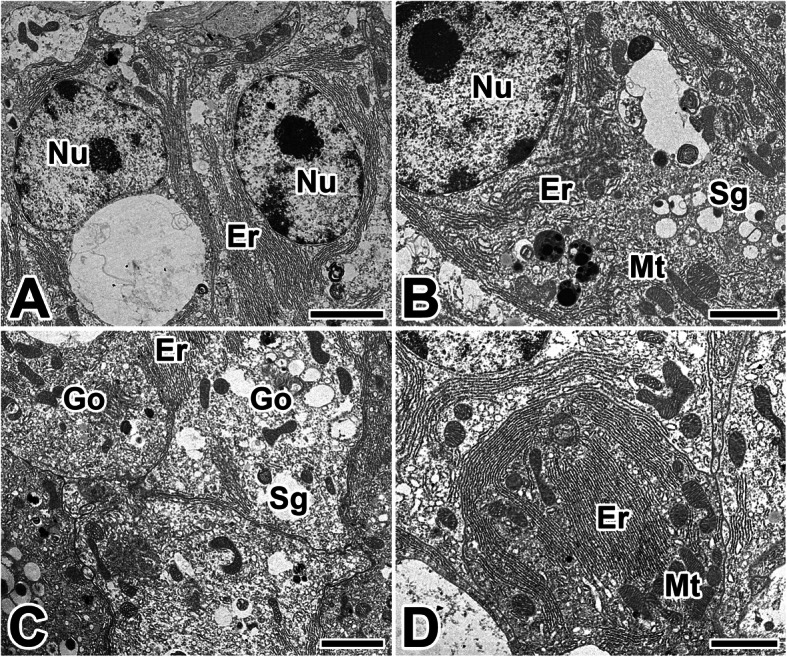
Transmission electron micrographs of the posterior silk gland in the caddisworm *H. nigrovittatus.***a** Glandular epithelial cells have a large nucleus (Nu) with a prominent nucleolus. **b**-**d** At the vicinity of secretory granules (Sg), large amount of rough endoplasmic reticulum (Er), mitochondria (Mt), and Golgi apparatus (Go) are seen. Scale bars indicate 5 μm (**a**), 2 μm (**b**), and 1 μm (**c**, **d**), respectively

At the posterior part of the silk glands, active production of the silk substances is observed. Since this glandular epithelial cell actively synthesizes and secretes silk precursors, each epithelial cell exhibits a productive cytoplasm containing a large nucleus with fine granular chromatin and a prominent nucleolus (Fig. 6a). Due to the abundant cisternae of rough endoplasmic reticulum (rER), the limiting membranes between adjacent cells cannot be easily distinguished (Fig. 6b).

The central part of the cell is occupied by large amounts of rER, mitochondria and nucleus. The Golgi apparatuses also appeared in the cytoplasm near the apical surface of the cytoplasm (Fig. 6c). The rER is uniformly distributed throughout the cell but is missing near the basal and apical borders. Small vesicles are synthesized from rER and secretory granules are formed by fusion with small vesicles from Golgi apparatus (Fig. 6d).

**Fig. 7:**
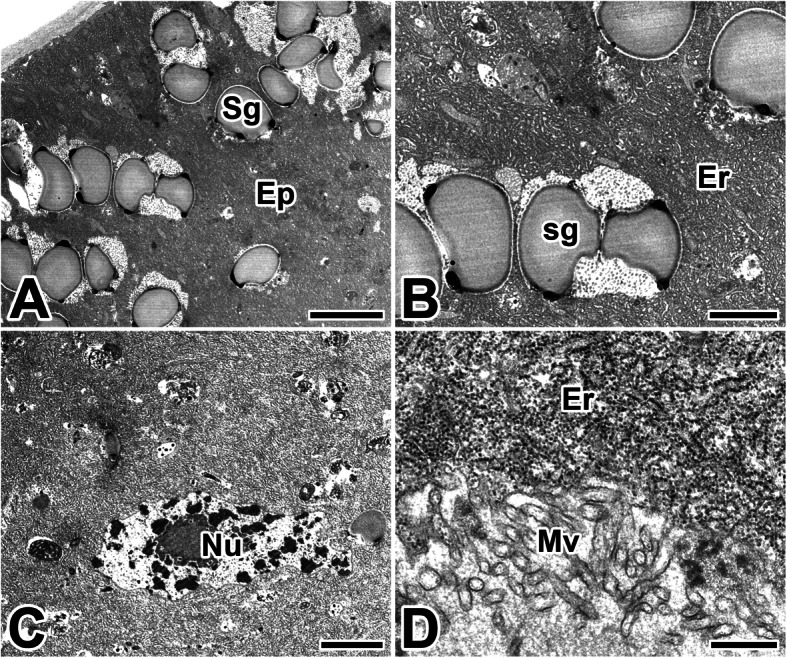
Transmission electron micrographs of the posterior region of the silk gland in the caddisworm *H. nigrovittatus.***a**, **b** Secretory silk products are visible as large secretory globules (Sg) in the epithelium (Ep). Er: rough endoplasmic reticulum. **c**, **d** Secretory products are transported across apical microvilli (Mv) by exocytosis, and they are released to the lumen as a major silk substance. Nu: nucleus. Scale bars indicate 5 μm (**a**), 2 μm (**b**), 5 μm (**c**), and 0.5 μm (**d**), respectively

The caddisworm silk glands store large quantities of silk dope within the lumen, and secretory products of the glandular epithelium are clearly visible as large granules contained within the basal epithelium of the posterior silk glands (Fig. 7a). The amount of fine globular granules is gradually increased during active production of silk. These granules frequently appear to disperse to form an amorphous, electron-lucent deposit (Fig. 7b).

The appearance of secretory vesicles varies from cell to cell and can be representative of the secretory cycle. There are several secretory vesicles in these cells, thus causing this area to stain much thinner (Fig. 7c). These fine granular materials appear to be transported across the apical membrane by exocytotic activity, and they can be added to the luminal secretory product as a major silk substance (Fig. 7d).

**Fig. 8:**
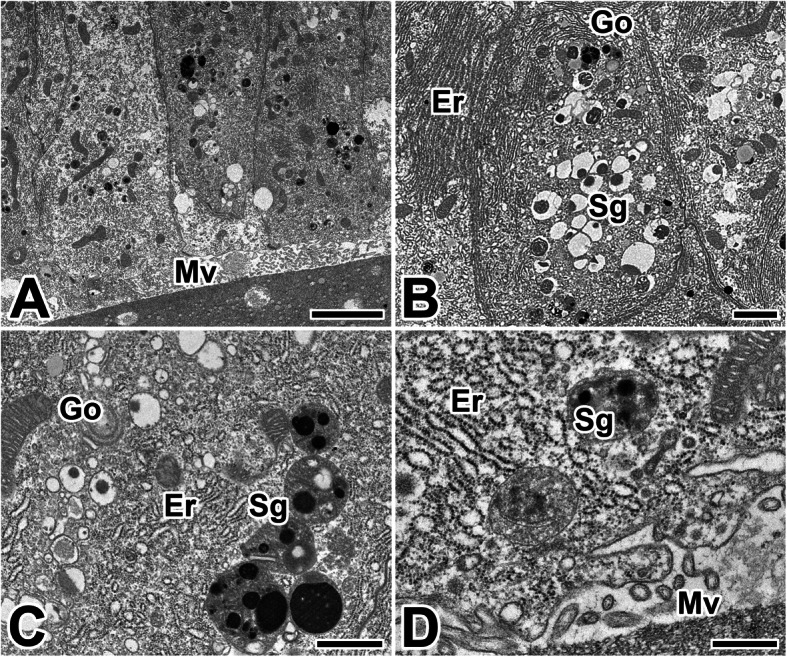
Transmission electron micrographs of the anterior silk gland in the caddisworm *H. nigrovittatus.***a** The adhesive products are produced as electron–lucent spherical granules (Sg). **b**, **c** In the cytoplasm, extensive rough endoplasmic reticulum (Er) and cisternae of Golgi apparatus are seen. Mv: microvilli. **d** The secretory granules are discharged into the lumen and then mixed to silk dope (Sd). Scale bars indicate 5 μm (**a**), 2 μm (**b**), 1 μm (**c**), and 0.5 μm (**d**), respectively

The silk gland of the anterior region secretes an adhesive substance that cements the two filaments together. The secretory granules, presumed to be precursor of the adhesive substance, appeared in the basal cytoplasm of this epithelium. These secretory products are accumulated in the apical cytoplasm of glandular epithelial cells as an appearance of electron-lucent spherical granules (Fig. 8a).

An extensive rER occupies the whole remaining space of the glandular epithelial cells, particularly near the nucleus. It can be also observed through transmission electron microscope that a Golgi apparatus and isolated vesicles of Golgi bodies are well developed (Fig. 8b). The amount of electron-dense material is gradually increased during active production of silk. These granules frequently appear to aggregate with some others to form a multivesicular body (Fig. 8c). With progressive maturation of silk products, these secretory granules are gradually discharged into the lumen via the apical microvilli (Fig. 8d).

**Fig. 9:**
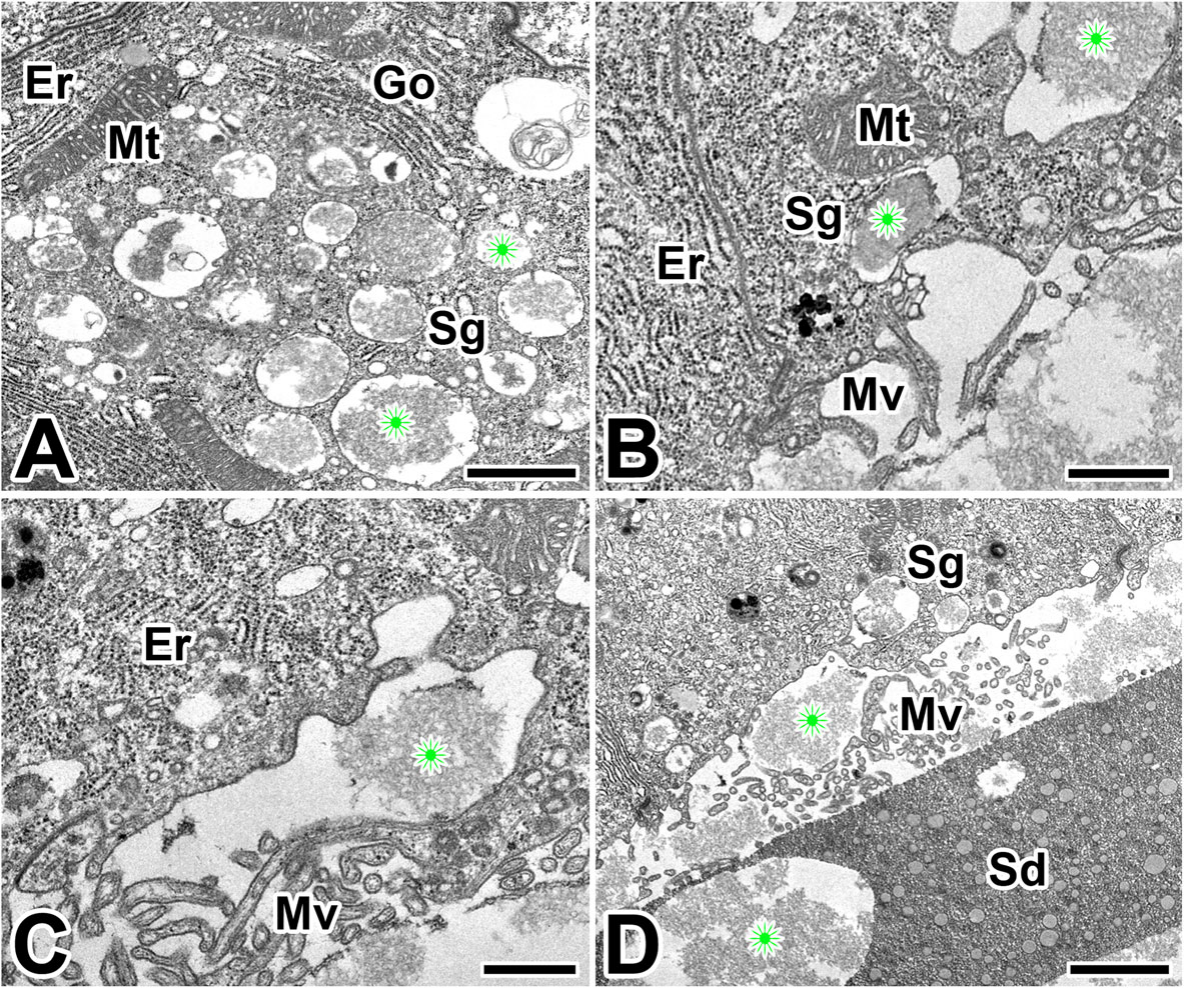
Transmission electron micrographs of the anterior region of the silk gland in the caddisworm *H. nigrovittatus.* Secretory granules (Sg) are electron-lucent spherical granules with fine fibrous materials (asterisks). At the vicinity of secretory granules well developed rough ER (Er), Golgi apparatus (Go), and mitochondria (Mt) are seen. D: Secretory granules are released to the lumen via the microvilli (Mv) by the merocrine secretion. Gradual transition of the secretory materials (asterisks) from cell to lumen are clearly seen. All scale bar indicates 0.5 μm (A)

During the production of adhesive substances in the anterior region of the silk gland, the small secretory vesicles are formed near the maturing face of Golgi apparatus. These Golgi vesicles condense to form secretory granules for releasing their contents by exocytosis. These secretory granules are approximately less than 0.5 μm in diameter, and are filled with some fine fibrous substances (Fig. 9a). After its synthesis is complete, these vesicles fuse with one and other to form larger granules that eventually move to the apical membrane and are exocytosed into the lumen as a secretory product (Fig. 9b).

The apical plasma membrane of glandular epithelium is collectively form the brush border to increase surface area. Short microvilli are formed as cell extensions from the plasma membrane surface in the area. The secretory material is frequently seen to be discharged into the lumen through the apical surface of the cells (Fig. 9c). Mature secretory granules accumulated at the apical cytoplasm are released to the lumen by the mechanism of merocrine secretion during the active process of silk production (Fig. 9d).

## Discussion

Silks are produced solely by arthropods, and only by animals in the classes Insecta, Arachnida, and Myriapoda. Silks are fibrous proteins containing highly repetitive sequences of amino acids and are stored in silk glands as a liquid and configure into fibers when spun at secretion (Craig 1997). Basically, two different processes of silk-producing systems are known among the different species of arthropod animals: terrestrial and aquatic silk productions (Ashton and Stewart 2019). In terms of molecular adaptation, aquatic caddisflies have diverged from a dry silk-spinning ancestors shared with terrestrial moths and butterflies (Stewart and Wang 2010). Indeed, except for some minor difference, the morphology and structure of the silk glands of caddisworm and silkworm were found to be very similar.

The caddisworms larva uses silk not only to produce capture nets to collect food particles from the water environment but also to construct silken cases for their shelters in running water. It has been reported that the caddisworm silk is mechanically well adapted as an underwater structural material (Ashton et al. 2016). The overall amino acid composition of wet spun caddisworm silk is strikingly different from dry spun silkworm silk (Ashton and Stewart 2019). The major structural component of caddisworm silk is high molecular weight H-fibroin (Stewart and Wang 2010), which like silkworm H-fibroin (Yonemura et al. 2009). In a similar process, orb-weaving spiders dry-spin their silks into air from liquid crystalline precursors stored at high concentration in silk glands (Vollrath and Knight 2001).

In the larva of caddisfly *H. nigrovittatus,* we can see that the adhesive silk ribbon is extruded out of the spinneret located at the apical end of the labium. Our SEM examination for the silk fiber shows the silk product looks like a flattened fibrous ribbon with a distinct peripheral coating on a core fiber. Although the silk material is produced by a pair of silk glands but they emerge from spinneret as a single ribbon. Previous report has shown that the mechanical stresses during spinning are known to orient the preformed fibrils (Engster 1976). When silk fibrils pass through the narrow passage from the glandular cavity to the spinneret, they may align in a linear fashion, being squeezed together and flattened to form an insoluble ribbon.

In this experiment, we can see that the function and morphology of the silk glands of the aquatic caddisworm (Trichoptera) and terrestrial silkmoth (Lepidoptera) are basically the same except for some minor histological differences. Silk gland of the larva of *B. mori* is a typical exocrine gland designed to secrete large amounts of silk proteins. Akai (1984) also observed that the secretory materials found in the silk gland are produced from three distinct regions: anterior, middle and posterior silk gland. Among them, the posterior silk glands secrete the gelatinous silk components - fibroin, which is the main silk component in the cocoons of the silk moth.

Ohkawa et al. (2014) purified a silk protein from caddisfly larva *Stenopsyche marmorata*, and Ashton et al. (2012) studied the morphology and biochemical traits of adhesive silk of aquatic caddisworms. They found that the silk glands of caddisworm form Z-type silk glands, which belong to complex glands according to the structural complexity. Our present study using TEM clearly reveals that the two different types of glandular substances were produced within the silk glands of caddisworm. Previous study reports the silk fibroin is produced in the posterior silk gland of the silkworm *B. mori*, while almost no silk fibroin is produced in the anterior part of the silk gland (Sasaki et al. 1981).

Furthermore, the silk proteins polymerize into a fiber during spinning when they pass through the anterior silk gland section and the spinneret (Sehnal and Sutherland 2008). It has been revealed recently that the silk fibers begin to form in the gland at a cuticular narrowing of the entrance leading to the spinneret, and the silk comprises a thin adhesive peripheral coating on a tough viscoelastic core fiber. In addition, the thin adhesive layer contains glycoproteins and a heme-peroxidase in the peroxinectin subfamily (Ashton et al. 2016). This is also consistent with our microscopic observation because a covering layer containing a gluey substance is seen in the complex structure of the silk ribbon in *H. nigrovittatus.*

Aquatic caddisfly larva also spins adhesive underwater silk with a repeating motif conserved in the phosphorylated H-fibroin (Stewart and Wang 2010). Phosphorylation of serines and the accumulation of basic residues in the silk proteins allowing underwater spinning of caddisworm. A fibrous adhesive underwater threads from marine amphipod *Crassicorophium bonellii* produces the filamentous silk as a mixture of mucopolysaccharides and protein deriving from glands representing two distinct types (Kronenberger et al. 2012). They reported the carbohydrate and protein silk secretion is dominated by complex β-sheet structures and a high content of charged amino acid residues.

Akai and Kobayash (1965) have shown by radioautography that glycine-^3^H, injected into the larvae of silkworm *B. mori* is accumulated within fibroin globules. From this observation, the silk fibroin accumulated within the cisternae of the ER is transported to the Golgi vacuoles via Golgi vesicles. Mature fibroin globules finally move towards the luminal space, and then secrete their content by a reversed pinocytotic process (Sasaki et al. 1981).

In this study, the fine structural characteristics obtained from the observation of cellular event during the production of silk substances within the glandular epithelium of caddisworm silk glands are consistent with that of terrestrial silkmoth *B. mori* (Sasaki et al. 1981; Akai 1984). However, the process of web-building spiders is quite different from caddisworm, since the secretory silks of spiders are solely produced by way of rough ER of each glandular epithelial cell with no relationship with Golgi apparatus. Unlike the typical silk production in *B. mori* via both rER and Golgi apparatus, the ampullate silk glands of spider seem to bypass either the concentrating or packaging steps by Golgi apparatus (Moon and Tillinghast 2002, 2004; Moon 2018).

In *H. nigrovittatus,* the silk gland also secretes an adhesive substance that cements the two filaments together. The secretory products are accumulated at the anterior region of each silk glands as a form of electron-lucent spherical granules. An extensive rER occupies also the whole remaining space of the glandular epithelial cells, and secretory granules are gradually released into the luminal space by the mechanism of merocrine secretion. The Golgi bodies also play an important role during the process of secretion. However, their contribution to gluey silk production seems to be very restricted since only a small agranular cisternae can be distinguished.

After being discharged, the silk dope within the lumen looks with two concentric layers. Silk product transported from the posterior region is evenly surrounded by a coating medium secreted from the anterior region of silk gland. Vollrath and Knight (2001) also found that the secretory part of the ampullate gland in orb-web spiders has two distinct zones. As the secretion of posterior zone flows towards the funnel, it is coated by a viscous homogeneous liquid secreted near the anterior zone. The major secretion of posterior zone is identified as spidroin, the major protein making up spider dragline silk (O'Brien et al., 1998). Whereas, the coat protein secreted at the anterior zone is identified as glycoprotein in the thread biochemically (Weiskopf et al. 1996).

Similar to caddisworms, it has been reported that glycoproteins produced from the aggregate silk glands in web-building spiders are to make the thread sticky (Vollrath and Tillinghast 1991; Crew and Opell 2006). This is a very interesting because an example of divergent evolution is found within the evolutionary history of silk production. Despite the variation between taxonomic groups, the high similarities of proteins between species suggests that they are highly important for the survival of silk-producing animals, in functioning as adhesive materials, necessary to capture prey using the adhesive silk fibers. It seems that the caddisflies also have evolved over millions of year to preserve their unique silk-producing system within the aquatic environment to overcome the risk of dilution to stickiness.

## Conclusion

We demonstrate the fine structural characteristic of the aquatic silk spinning system in the caddisworm, *Hydatophylax nigrovittatus.*

Although silk fibers are made under aquatic conditions, the cellular silk production system is quite similar to that of terrestrial arthropods.

Silk is produced by a pair of labial silk glands, and silk fibers begin to spin from the spinneret with a thin adhesive peripheral coating on a core fiber.

Silk production is achieved by two independent processes in the silk gland: synthesis of silk fibroin in the posterior region and production of adhesive glycoproteins in the anterior region.

At the cellular level, each substance of fibroin and glycoprotein is specifically synthesized at different locations, and then transported from the rER to the Golgi apparatus as transport vesicles, respectively.

Thereafter, the secretory vesicles gradually increase in size by vesicular fusion, and they are exocytosed into the lumen by a mechanism of merocrine secretion.

## Data Availability

Materials described in the manuscript, including all relevant raw data, will be freely available to any scientist wishing to use them for non-commercial purposes.

## Abbreviations

rER: Rough endoplasmic reticulum
TEM: Transmission electron microscopy
